# Development of an Artificial White Spot Lesion Creation Protocol: A Preliminary Study

**DOI:** 10.7759/cureus.60226

**Published:** 2024-05-13

**Authors:** Rajeletchmi Seevalingam, Norziha Yahaya, Alizae Marny F Syed Mohamed, Hetal A Kumar

**Affiliations:** 1 Department of Restorative Dentistry, Faculty of Dentistry, Universiti Kebangsaan Malaysia, Kuala Lumpur, MYS; 2 Department of Family Oral Health, Faculty of Dentistry, Universiti Kebangsaan Malaysia, Kuala Lumpur, MYS

**Keywords:** scanning electron microscope, durations, acetate buffer solution, demineralization, lesion depth, white spot lesion

## Abstract

Background and objectives: Protocols that determine the lesion depth of specific demineralization solutions are lacking. This in vitro study aimed to evaluate various lesion depths of artificial white spot lesions (WSLs) at different exposure times.

Materials and methods: Artificial WSLs were created by placing 30 extracted human premolar teeth into 0.05M acetate buffer solution with a controlled environment of pH 4.4 at 37ºC, which were then exposed in the solution for various durations of 4, 5, 6, 8, 10, and 12 days. The specimens were visually examined using the Ekstrand‐Ricketts‐Kidd (ERK) system to confirm the WSL, followed by buccolingual sectioning, and evaluated under a scanning electron microscope (SEM) to measure the lesion depth.

Results: The SEM showed that the mean lesion depths of representative specimens were 101.33 µm (day 4), 124 µm (day 5), 159 µm (day 6), 187 µm (day 8), 202 µm (day 10), and 212 µm (day 12). The artificial WSL was visually demonstrated in grades 1 and 2 of the ERK system.

Conclusions: The depths of the lesions increased as the duration increased from day 4 to day 12, indicating that the lesion depths increased with the more prolonged exposure to the acetate buffer solution.

## Introduction

A white spot lesion (WSL) is an active carious demineralization in the tooth's enamel. The demineralization process occurs when plaque accumulates on the tooth surface, which will harbor acidogenic bacteria such as *Streptococcus mutans* and *Lactobacilli*. The remineralization threshold decreases once the pH is lowered and when fermentable carbohydrates are present. Therefore, demineralization supersedes. This begins the initial carious process, also known as WSL formation.

This WSL surface is characterized by milky, white opacities on smooth surfaces of the tooth and is softer than the surrounding sound enamel. The whitish appearance is due to the optical phenomenon following the loss of enamel minerals. The degree of demineralization is directly proportional to the translucency of enamel, which has the highest refraction index (RI) compared to dentin and cementum [1]. The RI of hydroxyapatite in enamel ranges from 1.62 to 1.65, while the RI in the demineralized lesion is only 1.00 when air-dried. This decrease in the RI of enamel is due to the removal of air that fills the pores present in WSL when air is blown onto the surface [2].

Many studies use in vitro models to examine fundamental processes associated with enamel demineralization. Studies have reported various lesion depths to observe the visible WSLs in vitro [3,4]. Most studies do not measure the lesion depth or only estimate the depth of demineralization for lesions extending between the enamel and dentine without obvious cavitations or surface damage [5-7]. The observed differences may be due to the type of teeth used, such as bovine incisors, human premolars, or molars [5,8]. In addition, whether it is teeth with natural WSL [5,6,9] or teeth with created artificial WSL. Various demineralization solutions have been used to create artificial WSL [3,5,6,10,11]. In addition, instruments used to measure the lesion depth include a scanning electron microscope [3] and optical coherence tomography [12], which use different measurement software. Some studies include the evaluation of the WSL with various visual assessments such as the International Caries Detection and Assessment System (ICDAS), Ekstrand‐Ricketts‐Kidd system (ERK), Caries Assessment Spectrum and Treatment (CAST) [13-15], and Gorelick WSL score that is related to the area of the lesion [16].

The cause, size, and depth of the WSLs must be ascertained before providing treatment options to a patient, as treatment results will vary depending on the enamel substructure involved. The significant clinical appearance of the WSLs indicates the involvement of the lesion depth, which later leads to the management of the WSL. It is important to minimize the false-positive error in clinical situations, especially in occurrences of WSL, when caries is absent. Visual detection methods should be helpful in the decision of whether treatment should be provided.

Protocols for determining the lesion depth in specific demineralization solutions are lacking. Therefore, this in vitro study was conducted to create various depths of WSLs in relation to the duration of days using the acetate buffer solution.

## Materials and methods

Study design and sample size

This experimental in vitro study is part 1 of a main study on the WSL depth. Ethical approval was obtained from the Centre for Research and Instrumentation Management at the Universiti Kebangsaan Malaysia (UKM PPI/111/8/JEP-2021-771) prior to the commencement of this study.

This preliminary study did not intend to compare any differences between the depths of the lesions involved. Therefore, a predetermined sample size was not carried out. Moreover, due to the significant financial associated with the preparation of the specimen for scanning electron microscopy (SEM) and measurement, it was deemed sufficient to use two specimens for determining the lesion depth.

Fifty-two human premolar teeth extracted for periodontal or orthodontic reasons were collected from dental clinics at UKM. The teeth were cleaned under running water to eliminate any traces of blood or soft tissue. The teeth were then placed in a plastic container with distilled water. Later, each tooth was cleaned with an ultrasonic scaler (EMS® Piezon Master 100, Electro Medical Systems, Vaud, Switzerland) to remove any calculus and then disinfected with a 10% buffered formalin solution (LTC 2079 - 5L) for 24 hours to prevent dehydration. Finally, all samples were stored in distilled water at room temperature until used. The collected teeth were examined for any restorations, caries, stains, demineralization, fluorosis, or enamel defects on the buccal surface to be studied. Eight were excluded due to fluorosis and cracks.

Preparation of window

A mixture of freshly mixed polymethylmethacrylate (PMMA) of cold cure acrylic (Meliodent, Kulzer, UK) was prepared. The specimens were flushed with distilled water and placed in a silicone mold of 2 cm in diameter, and the whole linguistic part of the specimen was embedded in the acrylic mixture. Only the buccal part of the specimen was free from the acrylic. Once the mixture has hardened/set, a standardized round working window of 5 mm in diameter was pasted on the buccal surface of the specimen crown using sticker paper. This was followed by applying an acid-resistant nail varnish (Sephora, France) around the sticker.

Lesion formation and visual assessment

Thirty specimens were assigned identification numbers and divided into six groups (n=5 per group) for different exposure durations of 4, 5, 6, 8, 10, and 12 demineralization days. The round sticker was removed. The specimens were immersed in a 2.0 mL/mm^2^ solution of 0.05M of an acetate buffer solution adapted from the study by Kumar et al. [17]. The pH level of the solution was constantly monitored using a pH meter, and the specimens were incubated at pH 4.4 with a temperature of 37°C to ensure consistency. However, due to the possibility of ionic exchange, pH fluctuations may occur, necessitating the use of fresh acetate buffer.

After completion of the demineralization process according to the assigned durations, specimens were flushed with distilled water. All specimens underwent a visual assessment following the ERK system (Table 1) criteria. This ensured the visual presence of WSLs, and the assessment was tabulated in Microsoft Excel. Only specimens with a score of 1 and 2 were included in the study.

**Table 1:** Ekstrand‐Ricketts‐Kidd (ERK) system

Score	Criteria
0	No or slight change in enamel translucency after prolonged air drying of more than 5 seconds
1	Opacity or discolouration is hardly visible on wet surfaces but distinctly visible after air-drying
2	Opacity or discolouration distinctly visible without air drying
3	Localized enamel breakdown in opaque or discoloured enamel or greyish discolouration from the underlying dentine
4	Cavitation of opaque or discoloured enamel exposing dentine

Two independent restorative specialists (NY, HAK) trained and calibrated the examiner (RS) on the ERK system scores. Any discrepancies in the evaluation were resolved through mutual agreement. The visual assessment reliability of the examiner was evaluated on thirty (n = 30) specimens in the same room and under the same controlled lighting conditions. The first and second assessments were conducted 14 days apart to test for intra- and inter-examiner reliability with IBM SPSS Statistics for Windows, Version 26 (Released 2019; IBM Corp., Armonk, New York, United States). The intra-class correlation (ICC) indicates excellent reliability for both intra-examiner (0.868) and inter-examiner (0.965) evaluations.

Lesion scanning and depth measurement

Two specimens per group that fulfilled the criteria for scores 1 and 2 of the ERK system were sectioned using a high-speed precision cutting machine (Buehler Isomet 4000, Switzerland) at 1000 rotations per minute with a feed rate of 3.98 mm/min. After being cut into mesial and distal halves [3], the specimens were stored in distilled water.

The surface of each specimen from each group was gold-plated using a sputter coating machine (Emitech-K500X, Quorum Technologies, Ashford, UK). This was followed by examination under a scanning electron microscope (Field Emission SEM, SUPRA 55VP, Zeiss, Germany) with magnifications of x500 and x2000 to detect topographical changes and measure the WSL lesion depth.

Measurements were taken at the midpoint of the buccal curvature using Zeiss SmartTIFFV2 software (Carl Zeiss SMT, Germany). Two measurements were taken for each specimen; if there were any differences between them, a third reading was taken to discard the aberrant one. The mean (Excel, Microsoft 365, Microsoft Corp, Redmond, WA, USA) of the two closest measurements of lesion depth was used in the calculation [3]. Although multiple data-analytic modeling approaches were reviewed regarding outlier removal, this study only involves small and limited data of specimens [18]. No statistical analysis was involved, and removing the outlier measurement would not negatively impact the results. Therefore, this study protocol has implemented the removal of the outlier by excluding the measurement that deviates significantly from others. The deviation could be caused by imprecise instruments, environmental factors, or human error.

## Results

Eight specimens were observed to lack a WSL (score 0). The balance specimens were graded in scores 1 and 2 of the ERK system. Table 2 demonstrates the lesion depth observed in all 12 specimens following exposure to acetate buffer solution for various durations. The lesion depth increased with the duration from day 4 to day 12, indicating that the lesion depths increased with more prolonged exposure to the acetate buffer solution.

**Table 2:** Lesion depth (µm) of WSLs based on the days of exposure to the acetate buffer solution WSL: White spot lesion

Specimen	Day					
	4	5	6	8	10	12
	Average depth (µm)					
1	105.60	121.70	160.70	185.60	201.33	212.34
2	97.06	127.24	157.98	189.34	202.13	208.60
Mean	101.33	124.47	159.34	187.47	202.23	210.30

**Figure 1:**
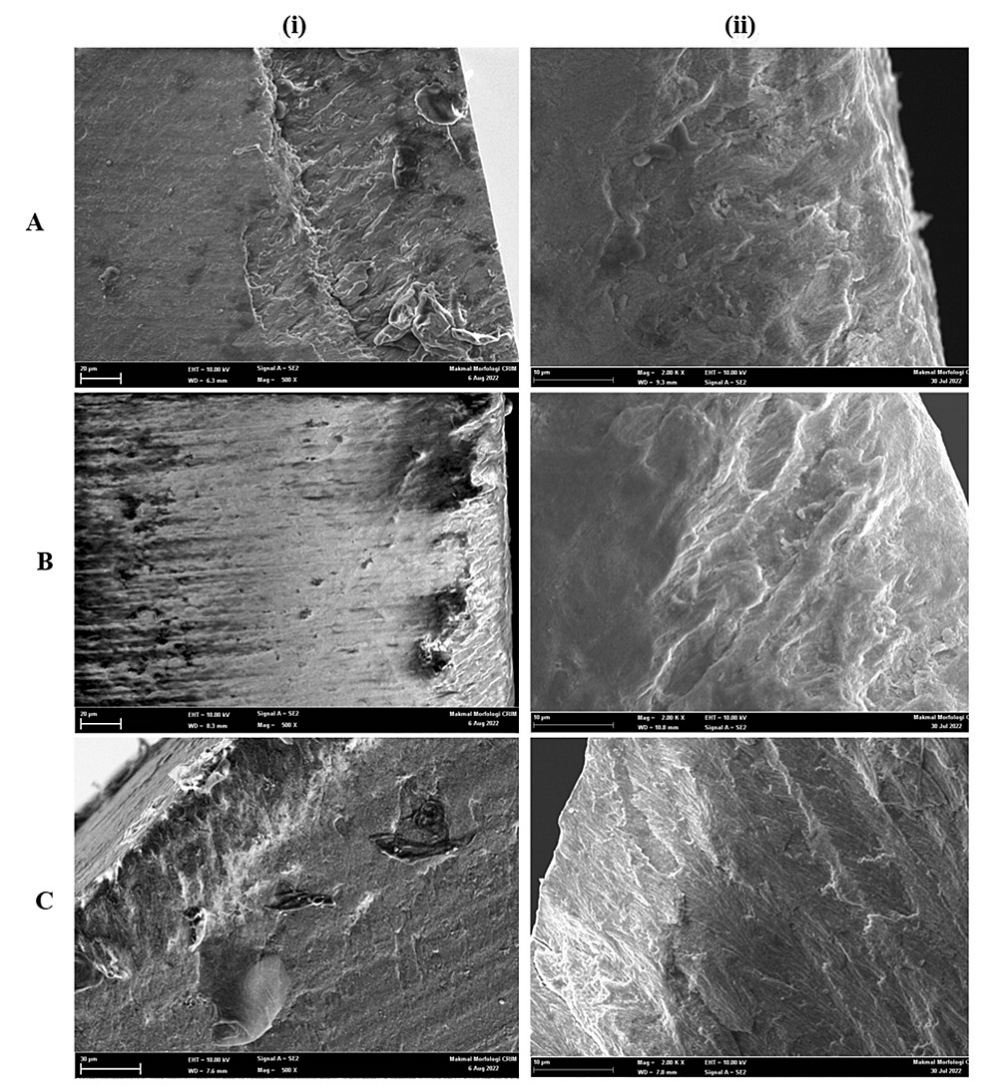
SEM observations revealed white spot lesions on the buccolingual cross-section A-4 days, B-5 days, and C-6 days (i) 500x and (ii) 2000x magnification SEM: Scanning electron microscopy

**Figure 2:**
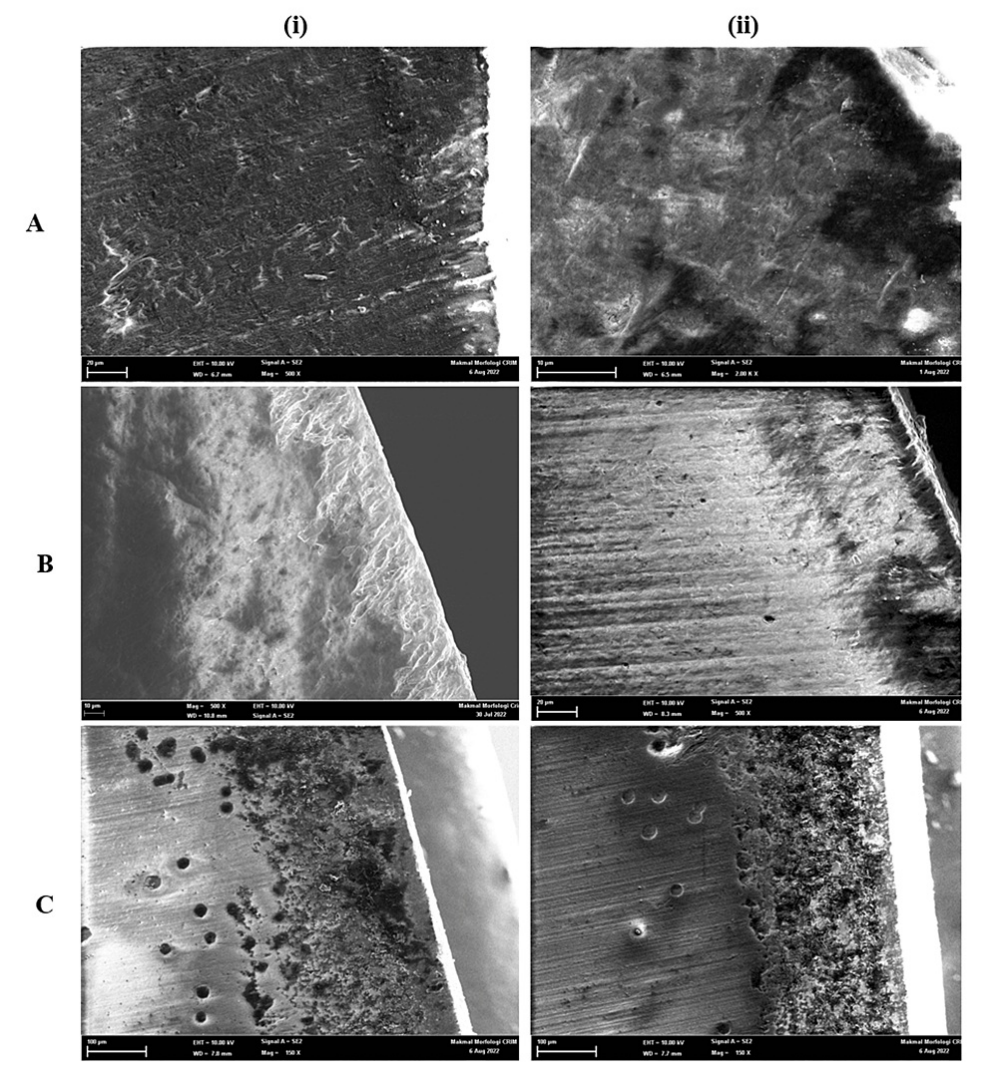
SEM observations revealed white spot lesions on the buccolingual cross-section A-8 days, B-10 days, and C-12 days (i) 500x and (ii) 2000x magnification SEM: Scanning electron microscopy

Figure 1 and Figure 2 show the SEM images of the representative specimens of the different experimental groups. The images show histological features and depths of the lesions according to the duration of exposure to the acetate buffer solution. The lesions extend from the tooth's surface to the demineralization front at an obtuse angle.

Figure 1A to Figure 1C indicate an increase in surface porosities. Inter and intra-prismatic microspores are enlarged. Figure 2A and Figure 2B demonstrate an increase in pores with demineralization of the prism center that resembles a honeycomb. Figure 2C shows demineralized enamel with an irregular, pitted, and discontinued surface. This appearance indicates the dissolution of the peripheral prism and a difference in the solubility of the mineral acid.

## Discussion

Healthy enamel is the most highly mineralized tissue in the organism, with 96% of its composition (by weight) represented by hydroxyapatite; the remaining 4% are organic fluids. Conversely, in a WSL, this mineral phase is diminished and replaced by organic fluids. This condition is the first stage of dental caries and is characterized by hypomineralization without any cavity formation. The prevalence of this condition has been reported to range between 10 and 49% [19], which is frequently seen in patients undergoing orthodontic treatment. The bacterial plaque accumulates around the brackets and the gingival area of the bands, which is pronounced especially in the cervical region of the tooth [20].

In general, white discoloration of enamel can be presented with differing degrees of clinical symptoms involving white appearance on the enamel [21]. The histology features occur via alternating phases of demineralization and remineralization, resulting in preferential dissolution and reprecipitation of WSL. The enamel is dissolved longitudinally and laterally along specific weak points of the crystalline structure. This creates pathways that are directly accessible from the external acid environment. In addition, subsurface demineralization causes pores at the center of the enamel prisms to enlarge while maintaining a relatively intact surface layer with a crystalline scaffold [22].

It is fundamental to detect and diagnose the WSL when it has only affected the outer layer of the tooth at the initial (non‐cavitated) severity levels. Earlier identification of lesions could provide patients with an opportunity for less invasive treatment with less destruction of tooth structure [14].

The ideal method for detecting WSLs should have high sensitivity (detecting disease when present) and specificity (confirming that disease is absent). Besides visual inspection that presented a good overall accuracy and high value for specificity, tactile probing, and digital photographic examination may also be useful [23,24]. The diagnosis of enamel defects can be performed through visual examination, considering the shape, size, color, and location of these defects [21]. The utilization of a well-established visual scoring system has been recommended as it can significantly increase the overall accuracy of the method in clinical practice alone, with no need for an adjunct method [13]. The advantages of this method are that it is simple to use, inexpensive, and clinically valid; its most significant disadvantage is that it is difficult to standardize [14]. Incorporating magnification into routine dental examinations by the general dentist can help in the early diagnosis and treatment of dental decay. This could prevent further progression of dental caries and reduce the incidence of tooth decay [25].

The opacities and the lesion on the enamel surface cannot be distinguished and will not be visible when the enamel is wet. The micropores on the surface filled with water have an RI of enamel of about 1.33, which is close to that of healthy enamel. Drying the tooth surface at least five seconds after being cleaned with pumice can enhance caries detection when visualized under adequate light compared to wet condition techniques [25]. After the air drying, the pores within the lesion will be filled with air, reducing RI to 1.0. Hence, the opaque enamel lesions become evident and distinct from the healthy enamel surface.

Most in vitro assessments have been shown for non-bracketed teeth. Limited in vitro studies still assessed the WSL with the presence of the orthodontic bracket. Studies have used cameras to detect and monitor the area and the fluorescence loss of WSL adjacent to orthodontic brackets, which resulted in a significant fluorescence loss influenced by the angle of camera position when the lesion is photographed [11,26]. Capturing images using this technique intra-orally can be challenging to standardize. The camera position will be limited by teeth rotations and angulations and the presence of wires, ligatures, or hooks. Even it will be limited by the soft tissue, especially the lips and buccal mucosa.

Studies have examined the response of WSLs to remineralization treatment. An OCT image was evaluated in an in vitro study of artificial demineralizing bovine teeth undergoing remineralization, which found a limit of remineralizing beyond 200 μm of the lesion depth [27]. On fluoride application or bioavailable calcium-realizing chewing gums, it was reported that a combination of 50-75 μm removal of the enamel surface followed by NaOCl treatment improved the mineral augmentation to 200 μm of the lesions. However, the mineral augmentation reduced and became unstable once deeper than 200 μm of lesion depth [28]. This highlights the significance of a WSL study beyond 200 μm of lesion depth.

In this study, the teeth were embedded in acrylic resin before demineralization instead of the opposite. This was to guarantee that the WSL prepared would be undisturbed or prevent any contamination or blockage of the prepared artificial WSL by the acrylic resin. There is limited information on how acrylic resin may affect the demineralization process and solution. Although the acrylic resin may stabilize the specimen during the cutting procedure, the specimens become dislodged from the acrylic resin on one occasion. This may be due to the cutting speed used. However, this highlights a concern about the need for retention between the specimen and the acrylic resin.

A non-invasive three-dimensional analysis method has been used to determine mineral density and characterize WSLs without cutting through enamel surfaces [29]. However, this technique is limited because it necessitates using extracted teeth and cannot be performed in a clinical setting. In addition, a significant correlation was demonstrated between the intensity of WSL color and lesion volume [30]. The depth and size of the lesion may affect the lesion color, with a larger lesion appearing whiter than a smaller lesion, potentially indicating greater enamel demineralization. Even this study found that scores 1 and 2 of the ERK system could not differentiate the depth of the lesion, as both scores were in a mixture in both lesion depths. This suggests that visual assessment alone may not accurately indicate the depth of enamel demineralization. Training and experience of clinicians may correlate the WSL depth with visual assessment.

Limitations and suggestions

Determining the methods for creating artificial WSLs is a challenging task, particularly when it comes to determining the depth of the lesions. Proper application of the correct amount of chemicals and precise timing are crucial when using acetate buffer solutions. Moreover, it is essential to address the issue of ionic saturation of the acetate buffer solution, as it can affect its effectiveness in producing demineralization. Buffers are solutions that resist changes in pH by donating or accepting H+ (hydrogen) ions. If the pH of a buffer goes out of range of buffering capacity, it will no longer be effective at maintaining a stable pH. Care should be given to applying this protocol in future studies. Further studies should be conducted to develop a standardized protocol for creating artificial WSLs with other demineralization materials.

It is worth noting that most indices used for detecting carious lesions focus on the occlusal surface [15] rather than the buccal or labial surfaces [16]. This is important for orthodontic patients since the WSL typically affects the buccal and labial areas of teeth. Since bracket attachments are usually placed in these areas, they become even more susceptible to WSLs. Future studies should focus on using the most reliable index, especially in a clinical setting, to provide a more realistic assessment of the depth of WSLs, taking into account challenges such as orthodontic bracket placement, plaque, tooth staining, and restorations. 

This study is the initial stage of the experimental research, specifically designed to precede part 2 of the main study. It was conducted on a smaller scale, and the results obtained were later applied to part 2 of the study. Since the objective of this study was to create lesion depths of 100 and 210 μm, only two specimens per duration group were selected for measurement. Two measurements were taken for each specimen, and the mean for each specimen was recorded. This was followed by calculating the mean of both specimens of each duration group. However, it may be recommended to include more specimens and increase the number of measurements to minimize potential bias and error.

## Conclusions

This study demonstrates the protocol development for creating artificial WSLs specifically for 100 and 210 μm lesion depths, specifically using the acetate buffer solutions. This protocol was later applied in part 2 of the main study. The lesion depths increased with prolonged exposure to acetate buffer solution from day 4 to day 12. Based on these findings, the protocol of exposure times of 4 and 12 days in the acetate buffer solutions was determined to be able to create WSLs with depths of 100 and 210 μm, respectively.

Clinically, it is important to determine the lesion depth, which appears to be potentially influenced in managing WSL. This study demonstrates the potential to enhance the application of specific lesion depth studies of WSL and infiltrant materials. Future studies will be able to aim to improve the clinical occurrence of WSLs, especially in managing lesion depths deeper than 200 μm.
